# Time-Varying Autoregressive Models: A Novel Approach Using Physics-Informed Neural Networks

**DOI:** 10.3390/e27090934

**Published:** 2025-09-04

**Authors:** Zhixuan Jia, Chengcheng Zhang

**Affiliations:** 1 School of Information Management, Wuhan University, Wuhan 430072, China; 2 Department of Health Sciences, Towson University, Towson, MD 21252, USA; czhang@towson.edu

**Keywords:** time-varying autoregressive model, physics-informed neural networks, generalized additive models, kernel smoothing, high-dimensional time series analysis

## Abstract

Time series models are widely used to examine temporal dynamics and uncover patterns across diverse fields. A commonly employed approach for modeling such data is the (Vector) Autoregressive (AR/VAR) model, in which each variable is represented as a linear combination of its own and others’ lagged values. However, the traditional (V)AR framework relies on the key assumption of stationarity, that autoregressive coefficients remain constant over time, which is often violated in practice, especially in systems affected by structural breaks, seasonal fluctuations, or evolving causal mechanisms. To overcome this limitation, Time-Varying (Vector) Autoregressive (TV-AR/TV-VAR) models have been developed, enabling model parameters to evolve over time and thus better capturing non-stationary behavior. Conventional approaches to estimating such models, including generalized additive modeling and kernel smoothing techniques, often require strong assumptions about basis functions, which can restrict their flexibility and applicability. To address these challenges, we introduce a novel framework that leverages physics-informed neural networks (PINN) to model TV-AR/TV-VAR processes. The proposed method extends the PINN framework to time series analysis by reducing reliance on explicitly defined physical structures, thereby broadening its applicability. Its effectiveness is validated through simulations on synthetic data and an empirical study of real-world health-related time series.

## 1. Introduction

The rapid expansion of time series data, fueled by the widespread adoption of digital technologies, the proliferation of connected devices, and the growing demand for real-time monitoring and decision-making, has traditionally been addressed using the Autoregressive (AR) model for temporal analysis and forecasting. At its core, the AR model asserts that future values of a time series are expressed as a linear combination of its past observations, contingent upon the essential assumption of stationarity, whereby the statistical characteristics of the series, such as its mean, variance, and autocovariance, remain invariant over time. This condition ensures model stability and the validity of inferential procedures; however, when applied to non-stationary data, AR models may produce biased or inconsistent results, thereby necessitating preprocessing techniques such as differencing or transformation to restore stationarity before model fitting. In real-world scenarios, the majority of applications and processes exhibit non-stationary dynamics, thereby violating the assumptions underpinning conventional AR models and often resulting in systematic bias and inconsistent parameter estimates [1,2]. Therefore, to adequately model the complex temporal dynamics inherent in non-stationary processes, it is imperative to adopt methodological frameworks that allow the evolution of statistical properties over time, either through parametric approaches with explicitly time-varying coefficients or through non-parametric techniques that flexibly adapt to local structural variations. Within this methodological paradigm, the Time-Varying Autoregressive (TV-AR) model serves as a well-established and theoretically grounded extension of the classical AR framework, providing a systematic means of capturing non-stationarity in time series data, as comprehensively detailed in [3]. Overall, the framework of the TV-AR model with *r* time lags can be expressed as

$$y^{\left(t\right)}=c^{\left(t\right)}+\sum_{k=1}^{r}\alpha_{k}^{\left(t\right)}y^{\left(t-k\right)}+\phi \left(t\right),$$

where $y^{\left(t\right)}$ denotes time series data, $c^{\left(t\right)}$ represents time-varying intercept, $\alpha_{k}^{\left(t\right)}$ are time-varying parameters, and perturbation ϕ(t) represents a zero-mean stationary process with $E\left[\phi {\left(n\right)}^{2}\right]=\sigma^{2}$, and E[ϕ(n)ϕ(m)]=0 for n≠m. In contrast to the standard AR model with fixed coefficients, the TV-AR model introduces temporally dynamic parameters, thereby offering substantially greater flexibility and adaptability for modeling non-stationary processes and capturing structural changes that frequently characterize empirical time series data. Further, in contexts where modeling complex interdependencies among multiple time series is essential and the assumption of independence across series is untenable, the Vector Autoregressive (VAR) model offers a powerful and theoretically well-grounded framework for multivariate time series analysis. As a multivariate extension of the univariate AR model, VAR jointly models multiple time series as functions of their own and each other’s past values, enabling comprehensive analysis of dynamic interactions, feedback effects, and temporal dependencies across variables. However, similar to the univariate case, the VAR model relies on the assumption of stationarity, which is frequently violated in empirical applications involving evolving systems. To address this limitation, the Time-Varying Vector Autoregressive (TV-VAR) model has been proposed as a more flexible multivariate extension, incorporating time-dependent coefficients that allow for the endogenous evolution of inter-variable relationships over time, thereby offering a more accurate representation of non-stationary processes [4]. Specifically, the TV-VAR model with *r* time lags can be expressed as

$$Y^{\left(t\right)}=C^{\left(t\right)}+\sum_{k=1}^{r}B_{k}^{\left(t\right)}Y^{\left(t-k\right)}+\Phi \left(t\right),$$

where vector time series data $Y^{\left(t\right)}\in R^{p}$, time-varying intercepts $C^{\left(t\right)}\in R^{p}$, time-varying matrix $B_{k}^{\left(t\right)}\in R^{p\times p}$, and Φ(t) are independent samples drawn from a multivariate zero-mean stationary process with covariance matrix Σ. Neural networks, as a cornerstone of deep learning, have demonstrated exceptional capabilities in approximating complex, nonlinear relationships and learn directly from data without relying on explicit model specifications, making them widely applicable across diverse domains. However, this data-driven flexibility often comes at the expense of interpretability, as neural networks generally function as “black boxes,” offering limited insight into their internal representations and decision-making mechanisms. To mitigate this, physics-informed neural networks (PINNs) have been proposed, embedding known physical laws, typically formulated as partial differential equations (PDEs), directly into the training objective by penalizing violations of governing equations, thereby ensuring physical consistency while reducing reliance on extensive labeled data [5]. Nonetheless, PINNs face inherent limitations due to their dependence on precise and well-posed prior knowledge of governing PDE formulations, which in practical applications are frequently unavailable, partially known, or suffer from ill-posedness, leading to challenges in model identifiability, stability, and robustness during training and inference. To enhance the applicability of PINNs in settings where governing physical equations are unknown or partially specified, we developed a hybrid framework that integrates PINNs with TV-AR and TV-VAR models. This integration exploits the expressive capacity and temporal adaptability of autoregressive structures to encode latent dynamics, while simultaneously imposing physics-inspired soft constraints through the PINN loss formulation. In addition, we benchmark the proposed approach against conventional TV-AR modeling techniques, including the Generalized Additive Model (GAM) and One-Sided Kernel Smoothing (OKS) methods, as introduced by [6], to evaluate its relative performance in capturing time-varying dynamics. The primary contributions of this research are summarized as follows:

- We propose a novel PINN-based time-varying autoregressive modeling framework, which integrates the time-varying constraints with neural network-based function approximation to capture complex, non-stationary, and dynamic temporal dependencies.
- We conduct comprehensive empirical evaluations using both synthetic datasets—designed to simulate smooth and abrupt regime shifts across different lag orders (r=1,2), and a real-world time series dataset, to systematically assess the performance and generalizability of all modeling frameworks. The comparison includes multiple evaluation metrics such as root mean squared error and trajectory reconstruction fidelity.
- We conduct a comprehensive comparative analysis of the PINN-based framework, focusing on its practical effectiveness in capturing dynamic temporal dependencies under non-stationary conditions. Specifically, we evaluate and discuss its strengths and limitations against GAM- and OKS-based methods and the stationary VAR model across several technical dimensions, including predictive accuracy in high-frequency regimes, robustness to structural breaks, and interpretability of time-varying coefficients.
- We release an open-source implementation of our PINN-based framework, developed using TensorFlow 2.18 for neural network training and SciPy for optimization and numerical solvers. The code is modular and extensible, supporting multiple configurations of lag structure, activation functions, training schedules (e.g., Adam, L-BFGS), and physical loss weighting schemes. Detailed examples and documentation are included to support reproducibility and ease of adoption.

The structure of the paper is organized as follows: Section 3 introduces the foundational concepts of the GAM- and OKS-based approaches and details the architecture of the proposed PINN-based framework. Section 4 presents simulation results across four distinct scenarios, covering both one-dimensional Time-Varying Autoregressive models and two-dimensional Time-Varying Vector Autoregressive models. Finally, Section 5 validates the effectiveness of our approach using a real-world health-related time series dataset.

## 2. Literature Review

Physics-informed neural networks (PINNs), introduced in 2019 [7], offer a mesh-free and data-efficient framework that embeds governing physical laws directly into the loss function via automatic differentiation, thereby enforcing both data fidelity and physical consistency, making them particularly well suited for solving ordinary and partial differential equations (ODEs/PDEs) in scenarios with sparse or noisy data. Since their inception, PINNs have been applied across a wide range of scientific domains. For instance, ref. [8] solved nonlinear PDEs such as the wave equation and the KdV-Burgers equation, demonstrating PINNs’ ability to model dispersive and smooth wave propagation. Additionally, ref. [9] addressed the fractional Fokker–Planck–Levy equation by integrating PINNs with classical finite difference methods, showcasing the potential of hybrid approaches for handling complex fractional dynamics. In fluid dynamics, PINNs have been employed to solve the incompressible Navier–Stokes equations with temperature coupling [10], model complex subsurface flow [11], and estimate hydrodynamic pressure distributions in journal bearings under both static and dynamic conditions [12]. A related study [13] further examined the forward and inverse processes of PINNs within the framework of the diffusive wave model. Next, in the context of stochastic systems modeling, ref. [14] extended PINNs to time-dependent stochastic PDEs by incorporating Monte Carlo sampling to represent uncertainties in initial and boundary conditions, while ref. [15] further advanced the framework by applying it to stochastic advection–diffusion–reaction systems, enabling the modeling of complex dynamics driven by random influences. To address model uncertainty and measurement noise, ref. [16] introduced Bayesian PINNs, incorporating variational inference to estimate posterior distributions over neural network parameters. Beyond conventional physical systems, PINNs have been increasingly adopted in specialized and interdisciplinary domains. For instance, ref. [17] utilized PINNs to solve neutron diffusion equations in nuclear reactor simulations, demonstrating their capacity to handle complex transport phenomena in nuclear engineering. Similarly, ref. [18] applied PINNs to characterize inhomogeneous wave velocity fields derived from ultrasonic experiments on mortar and glass specimens, highlighting their utility in materials testing and nondestructive evaluation. In the field of optical communications, ref. [19] employed PINNs to investigate lowest-order fiber modes and to simulate C+L-band optical systems by solving the paraxial Helmholtz equation and the Raman scattering evolution equation. Extending the framework further, ref. [20] proposed a physics-informed recurrent neural network incorporating a fractional-order gradient to enable rapid and accurate estimation of battery degradation in operational electric vehicles, while ref. [21] applied it to dynamical systems under ordinary differential equation constraints. In addition, materials analysis represents another domain where PINNs have been widely applied, including memristive synapse design [22] and the prediction of metallic material properties [23]. Collectively, these diverse applications underscore the versatility and robustness of PINNs as a powerful tool for learning and solving complex real-world systems governed by differential equations.

The Time-Varying (Vector) Autoregressive (TV-AR/TV-VAR) model, a pivotal extension of the classical AR/VAR framework, incorporates dynamically evolving coefficients to effectively capture regime shifts, structural breaks, and non-stationary behaviors in complex temporal systems, and has been widely applied across a broad range of disciplines. In health-related sciences, TV-AR models have proven valuable for tracking dynamic patterns and abrupt shocks, particularly in infectious disease surveillance and the construction of cross-regional transmission networks [24], as well as for producing accurate forecasts and conducting multi-impact assessments of the COVID-19 pandemic [25,26,27]. For general linear time-invariant systems, various algorithms have been proposed, including a gradient-based two-stage estimation framework [28], an accelerated gradient descent algorithm [29], a Taylor series-based gradient descent algorithm [30], and a weighted multi-innovation forgetting factor gradient algorithm [31]. In neuroscience, ref. [32] leveraged wavelet-based expansions of autoregressive coefficients to estimate TV-VAR processes in functional magnetic resonance imaging data, while ref. [33] applied TV-AR models to approximate multiwavelet basis functions for EEG signal classification. In psychology, TV-VAR models have been utilized to analyze emotional dynamics, with the authors of [34,35] uncovering subtle changes in affective states and identifying periods of increased emotional inertia and reactivity. Beyond biomedical and psychological applications, TV-AR/TV-VAR models have also been extensively used in economics, finance, and signal processing. For example, ref. [36] investigated herd behavior in China’s renewable energy sector using time-varying methods, while ref. [37] examined the dynamic interplay between the Infectious Disease Equity Market Volatility Tracker and Latin American financial markets. In the context of digital assets, ref. [38] modeled the time-varying behavior of Bitcoin prices, and ref. [39] analyzed daily stock market trading data from RCEP member countries. Additionally, ref. [40] introduced a time-varying first-order mixture integer-valued threshold autoregressive model driven by explanatory variables to capture fluctuations in daily trading volumes of VOW stock.

Overall, the integration of deep learning techniques into Autoregressive models has been extensively explored in recent research. For instance, ref. [41] proposed a hybrid model that combines discrete wavelet transform, seasonal autoregressive integrated moving average, and long short-term memory (LSTM) networks to effectively capture different components in the power time series of an offshore wind turbine in Scotland. In another study [42], the authors introduced a novel Deep Autoregression Feature-Augmented Bidirectional LSTM architecture specifically designed for time series forecasting. Similarly, ref. [43] developed a hybrid deep learning framework incorporating LSTM, temporal convolutional networks, and transformer architectures to enhance earthquake prediction accuracy. In the domain of energy economics, ref. [44] integrated ARIMA with LSTM to forecast crude oil prices, demonstrating the strength of hybrid models in capturing both linear and nonlinear dynamics. While these studies report strong predictive performance, they also underscore a persistent limitation: the lack of interpretability in traditional deep learning frameworks. To address this challenge, PINNs embed physical constraints into the learning process, thereby substantially improving model interpretability and explanatory power. However, despite their conceptual appeal, PINNs face notable challenges, most prominently, a strong reliance on high-quality, well-structured datasets. As highlighted by [45,46,47,48], the limited availability of such data can severely constrain their potential for real-world applications. Given the persistent difficulty in acquiring clean and comprehensive datasets, a promising direction may involve accepting a modest trade-off in transparency to broaden their applicability while retaining a meaningful degree of physical interpretability.

## 3. Proposed Methods for Time-Varying Autoregressive Modeling

Recall that the Time-Varying Autoregressive (TV-AR) model and Time-Varying Vector Autoregressive (TV-VAR) model with *r* lags can be expressed as

(1) $$\begin{array}{cc} y^{\left(t\right)} & =c^{\left(t\right)}+\sum_{i=1}^{r}\alpha_{i}^{\left(t\right)}y^{\left(t-i\right)}; \end{array}$$

(2) $$\begin{array}{cc} Y^{\left(t\right)} & =C^{\left(t\right)}+\sum_{i=1}^{r}B_{i}^{\left(t\right)}Y^{\left(t-i\right)}, \end{array}$$

where $Y^{\left(t\right)}\in R^{h}$, $y^{\left(t\right)}$ represents the multivariate/one-dimensional time series at time *t*, and each coefficient matrix $B_{i}^{\left(t\right)}\in R^{h\times h}$ captures the dynamic dependencies at lag *i*. Due to the high dimensionality of $Y^{\left(t\right)}$, jointly estimating all its components can be computationally intensive and statistically unstable. To address this, a common strategy is to decompose the estimation task by modeling each component $y_{j}^{\left(i\right)}$ individually:

(3) $$\begin{array}{c} y_{j}^{\left(i\right)}=C_{j}^{\left(t\right)}+\sum_{i=1}^{r}B_{j,i}^{\left(t\right)}Y^{\left(t-i\right)}. \end{array}$$

where $C_{j}^{\left(t\right)}\in R$ and $B_{j,i}^{\left(t\right)}\in R^{1\times h}$ denote the time-varying intercept and coefficient vector for the *j* component, respectively. In this section, we first introduce two traditional approaches for modeling the TV-AR and TV-VAR frameworks: the Generalized Additive Model (GAM-based) and One-Sided Kernel Smoothing (OKS-based). We then present a novel method based on physics-informed neural networks (PINN-based) to model these processes.

### 3.1. Generalized Additive-Based Method

The GAM is a flexible extension of linear models that replaces fixed coefficients with smooth, non-parametric functions, known as the basis, to capture nonlinear relationships between predictors and the response. Compared to traditional linear models, GAM offers greater flexibility and often yields improved predictive accuracy in real-world settings where the relationships between variables are nonlinear. Overall, the main structure of GAM with *n* basis functions is defined as

$$g\left(t\right)=\sum_{i=1}^{n}\zeta_{i}f_{i}\left(t\right),$$

where $f_{i}$ represents a smooth basis function and $\zeta_{i}$ represents the corresponding linear coefficient in the GAM framework. Next, when modeling the model 1 with *r* lags, estimating the time-varying coefficient vector using GAM is equivalent to solving the following optimization problem:

(4) $$\begin{array}{c} \beta =\underset{x,h^{T}x\leq 1}{\;argmin:\;}||y-{Vx||}_{2}^{2}. \end{array}$$

where $y=\left[y^{\left(m\right)},\ldots ,y^{\left(r\right)}\right]\in R^{m-r}$ denotes the time value vector with *r* lags; the constraint to avoid exploding values in the covariance function is h=[0,1,…,1]. The *i* row of the lagged time series matrix in GAM is given by

$$v_{i}=\left[\zeta_{0,1}f\left(i\right),\ldots ,\zeta_{0,n}f\left(i\right),\zeta_{1,0}f\left(i-1\right)y^{(i-1}),\ldots ,\zeta_{r,n}f\left(i-r\right)y^{\left(i-r\right)}\right]\in R^{n(r+1)},$$

where f(i) denotes a basis function evaluated at time *i*. Similar to TV-AR, solving the *i* time-varying coefficient matrix across all lags in the TV-VAR process 3 is equal to solving

(5) $$\begin{array}{cc} \; & B_{i}=\underset{x,h^{T}x\leq 1}{\;argmin:\;}\left|\right|y_{i}-Ax{\left|\right|}_{2}^{2}. \end{array}i$$

where $y_{i}=\left[y_{i}^{\left(m\right)},\ldots ,y_{i}^{\left(r\right)}\right]\in R^{m-r}$ represents the *i* time value vector; the constraint to avoid exploding values in the covariance function is h=[0,…,0,1,…,1]. The *k* row of the lagged vector time series matrix in GAM is given by

$$a_{k}=\left[\zeta_{0,1}f\left(k\right),\ldots ,\zeta_{0,n}f\left(k\right),\zeta_{1,1}f\left(k-1\right)y_{1}^{\left(k-1\right)},\ldots ,\zeta_{r,n}f\left(k-r\right)y_{p}^{\left(k-r\right)}\right]\in R^{\left(n(pr+1)\right)},$$

where f(k) represents the same interpretation as in the TV-VAR model. Next, a key challenge in GAM is selecting the appropriate type and number of basis functions, with options including cubic splines, P-splines, B-splines, and thin plate splines. Once the basis functions are chosen, various strategies can be employed to solve the optimization problems 4 and 5. While standard regression methods like Lasso and Ridge efficiently address unconstrained problems via convex optimization, they are less effective when explicit constraints (e.g., bounded parameters) are present. In such cases, gradient-based methods (e.g., projected gradient descent, Lagrangian techniques, and constrained quasi-Newton algorithms) offer a more principled framework, enabling the seamless integration of complex constraints into model estimation while preserving scalability and flexibility.

### 3.2. Kernel Smoothing-Based Method

**Figure 1:**
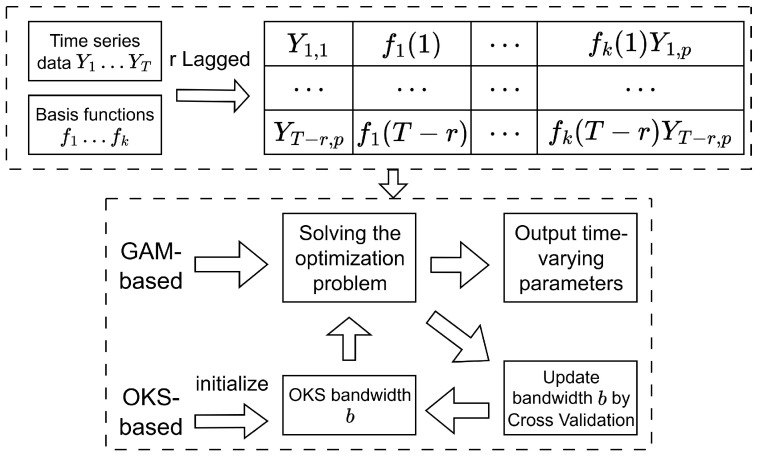
Workflow of the Generalized Additive Model and Onsided-Kernel Smoothing.

In the Kernel Smoothing (KS) framework, time-varying coefficients are estimated by locally fitting models at each target time point and subsequently integrating these estimates to construct smooth coefficient trajectories. Analogous to GAMs, KS methods rest on the foundational assumption that the effects of covariates can be represented through smooth, non-parametric functions. Typically, the KS weight at time $t^{*}$ is given by

$$K_{b}\left(t,t^{*}\right)=\frac{1}{\sqrt{2\pi b^{2}}}e^{-\frac{{\left(t-t^{*}\right)}^{2}}{2b^{*}}},$$

where *t* denotes a time point in the series, $t^{*}$ is the target time at which the time-varying parameter matrix is to be estimated, and *b* is the bandwidth parameter that controls the degree of smoothness in the estimation. In standard Kernel Smoothing (KS), estimating parameters at $t^{*}$ may inadvertently incorporate future data $\left(t>t^{*}\right)$, introducing look-ahead bias in predictive settings. To address this, One-Sided Kernel Smoothing (OKS) restricts estimation to past observations by setting $t^{*}=T$, the final time point, thereby ensuring temporal causality. The corresponding optimization problem is formulated as

(6) $$\begin{array}{cc} \; & \underset{\beta_{i,t^{*}}}{minimize:}\sum_{t=r}^{t^{*}}K\left(t,t^{*}\right){\left(y_{i}^{\left(t\right)}-\beta_{i,t^{*}}^{T}z_{t}\right)}^{2};\\ \; & Subject\;to:\;h^{T}\left|\beta_{i,t^{*}}\right|\leq 1. \end{array}$$

where $z_{t}={\left[1,y_{1}^{\left(t-1\right)},y_{2}^{\left(t-1\right)},\ldots ,y_{p}^{\left(t-r\right)}\right]}^{T}\in R^{\left(rp+1\right)}$, $\beta_{i,t}={\left[C_{i,t},B_{i,t,1,1},\ldots ,B_{i,t,p,r}\right]}^{T}\in R^{\left(rp+1\right)}$. Next, similar to the GAM approach, solving the optimization problem 6 is also equal to solving

(7) $$\begin{array}{c} B_{i}=\underset{x,\;h^{T}x\leq 1}{\;argmin:\;}\left|\right|d_{i}-Ax{\left|\right|}_{2}^{2}. \end{array}$$

where $d_{i}={\left[K\left(t^{*},t^{*}\right)y_{i}^{\left(t^{*}\right)},\ldots ,K\left(r,t^{*}\right)y_{i}^{\left(r\right)}\right]}^{T}\in R^{t^{*}-r}$ is the *i* time-indexed response vector, weighted by the kernel function. The corresponding row of the time-varying matrix *A* is defined as

$$a_{i}=\left[\sqrt{K(r+i-1,t^{*})},\sqrt{K(r+i-1,t^{*})}y_{1}^{\left(t^{*}-1\right)},\ldots ,\sqrt{K(r+i-1,t^{*})}y_{p}^{\left(t^{*}-r\right)}\right]\in R^{rp+1},$$

and the coefficient bound vector is $h=\left[0,1,\ldots ,1\right]\in R^{pr+1}$. A key component of the OKS method is the selection of bandwidth *b*. Larger bandwidths improve smoothness but risk underfitting, while smaller values may lead to overfitting by capturing noise. To address this, cross-validation is commonly employed to select an optimal b, as it effectively balances bias–variance trade-offs and adapts to diverse data structures via empirical risk minimization. The resulting optimization is typically solved using numerical techniques akin to those in the GAM framework. Finally, the workflows of both GAM- and OKS-based TV-AR/TV-VAR models are summarized in Figure 1.

### 3.3. The Physics-Informed Neural Network-Based Method

In the modeling of non-stationary processes, neural networks offer several notable advantages, including the ability to capture complex nonlinear relationships, handle high-dimensional inputs, and maintain computational efficiency, making them particularly well suited for nonlinear regression and highly time-varying systems. However, a common criticism of traditional neural networks is their lack of interpretability, which can reduce the reliability and transparency of the results in scientific applications. To address this, recent advancements, physics-informed neural networks (PINNs), have emerged, offering a more principled integration of domain knowledge into neural architectures by embedding physical laws or structural constraints directly into the learning process, thereby enhancing both model fidelity and interpretability. Typically, the PINNs are specifically designed to solve partial (ordinary) differential equations (PDEs/ODEs) by incorporating physical constraints directly into the learning process, and their methodological procedure can be summarized as follows:

- Define Physical Constraints: Formulate the mathematical model of the physical system, including the governing PDEs (or ODEs) and the associated initial and boundary conditions, which together define the structure of the solution space and guide the learning process.
- Initialize the Neural Network: Construct a neural network with randomly initialized weights and biases, where the network takes spatial or temporal variables as input and outputs the solution approximation.
- Construct the Loss Function: Define a composite loss function that penalizes deviations from the specified physical constraints, typically expressed as
  $$L_{total}=L_{PDSs}+L_{initial}+L_{boundary},$$
  where each term measures the discrepancy between the network’s output and the corresponding physical condition.
- Train the Network: Optimize the network parameters by minimizing the total loss function, typically using gradient-based optimization algorithms, such as L-BFGS-B and the conjugate gradient method.
- Prediction and Evaluation: After training, the neural network can be considered as a mesh-free, continuous surrogate for the solution, allowing for efficient evaluation across the domain by inputting the independent variables.

**Figure 2:**
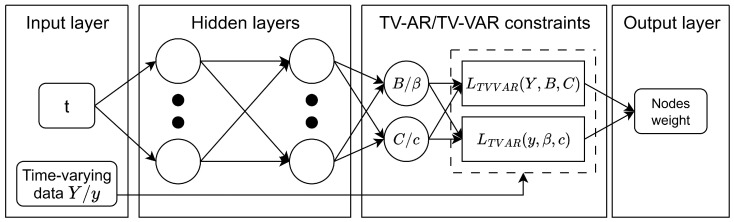
The main structure for the PINN-based time-varying (vector) autoregressive modeling approach.

Typically, The architecture of PINNs consists of three key components: (1) a sequence of *k* layers, each associated with an activation function, denoted by $F=[f_{1},\ldots ,f_{k}]$; (2) a set of weight matrices $W=[W_{1},\ldots ,W_{k}]$; and (3) a corresponding set of intercept vectors $b=[b_{1},\ldots ,b_{k}]$. The learning process in the PINN framework involves optimizing these weights and biases to minimize a composite loss function that encodes both data fidelity and physical constraints. Formally, this can be expressed as the following optimization problem:

(8) $$W,b=\underset{W,b}{\;argmin:\;}L_{total}\left(Y,W,b\right)$$

where $L_{total}\left(Y,W,b\right)$ denotes the total loss function incorporating both the data-driven and physics-informed components. Specifically, the structural constraints in TV-AR and TV-VAR models cannot be directly expressed as physical laws, making them less compatible with the standard framework of PINNs. This limitation arises because the model coefficients vary over time, preventing the entire system from being expressed as a single, time-invariant differential or algebraic equation. On the other hand, traditional deep learning-based approaches in time series analysis focus on directly estimating or forecasting the observed trajectories $Y^{\left(t\right)}$, as seen in applications like long-term structural health monitoring [49] and neurodegenerative disease modeling [50]. However, these end-to-end models often struggle to disentangle intrinsic temporal dynamics from noise or exogenous influences and tend to overlook the foundational mathematical structure of autoregressive processes, which are essential for interpretable and stable modeling of dynamic systems. This limitation becomes even more pronounced in time-varying autoregressive settings, where accurately capturing the evolution of coefficient matrices $B^{\left(t\right)}$ and intercept vectors $C^{\left(t\right)}$ over time is critical yet challenging. Therefore, in the context of time-varying processes, a more principled modeling strategy should prioritize learning the time-dependent coefficient matrices $B^{\left(t\right)}$ and intercept vectors $C^{\left(t\right)}$ rather than directly modeling the observed series $Y^{\left(t\right)}$, as these components more accurately characterize the system’s evolving dynamics, enable structural identification, and capture underlying temporal dependencies. From this perspective, the loss function of TV-VAR(r) at time *t* can be expressed as follows:

$$L_{TVVAR}\left(Y_{j}^{\left(t\right)},C^{\left(t\right)},B^{\left(t\right)}\right):={∥Y_{j}^{\left(t\right)}-C_{j}^{\left(t\right)}-\sum_{k=1}^{r}B_{k,j}^{\left(t\right)}Y^{\left(t-k\right)}∥}_{2}^{2}.$$

where $Y_{j}^{\left(t\right)}$ denotes the *j* element of the time series vector at time *t*, $C_{j}^{\left(t\right)}$ is the time-varying intercept term, and $B_{k,j}^{\left(t\right)}$ represents the time-varying coefficient row vector corresponding to lag *k* at time *t*. Similar, the TV-AR(r) can be expressed as

$$L_{TVAR}\left(y^{\left(t\right)},c^{\left(t\right)},\beta^{\left(t\right)}\right):={∥y^{\left(t\right)}-c^{\left(t\right)}-\sum_{k=1}^{r}\beta_{k}^{\left(t\right)}y^{\left(t-k\right)}∥}_{2}^{2}.$$

where $y^{\left(t\right)}$ is the time series data at time *t*, $c^{\left(t\right)}$ denotes the time-varying intercept, and $\beta_{k}^{\left(t\right)}$ represents the time-varying coefficient associated with lag *k* at time *t*. The overall structure of this approach is illustrated in Figure 2.

Next, to simplify the notation and align with the neural network architecture, we define the augmented lagged input vector for the TV-VAR model as $z_{t}={\left[1,Y_{1}^{\left(t-1\right)},\ldots ,Y_{p}^{\left(t-r\right)}\right]}^{T}$ denote the augmented lagged vector for the TV-VAR model, and for the TV-AR model as $g_{t}={\left[1,y^{\left(t-1\right)},\ldots ,y^{\left(t-r\right)}\right]}^{T}$. Using these definitions, the loss function for the proposed framework is formalized in Equation (9).

(9) $$\begin{array}{ccc} \; & L_{TVVAR}={∥y_{j}^{\left(t\right)}-F{\left(W,b,t\right)}^{T}z_{t}∥}_{2}^{2}; & L_{TVAR}={∥y^{\left(t\right)}-F{\left(W,b,t\right)}^{T}g_{t}∥}_{2}^{2}. \end{array}$$

To address the optimization problem presented in Equation (8) with the time-varying loss function defined in Equation (9), various gradient-based optimization methods can be employed, including the Broyden–Fletcher–Goldfarb–Shanno (BFGS) algorithm, the conjugate gradient method, etc. In this study, we employ the Limited-memory Broyden–Fletcher–Goldfarb–Shanno with Bound Constraints (L-BFGS-B) algorithm due to its demonstrated effectiveness in large-scale optimization, offering reduced memory overhead via limited-memory, fast convergence in smooth and differentiable loss landscapes, and explicit support for bound constraints, which are particularly well suited for estimating time-varying coefficients. Another crucial consideration lies in the design of neural network architectures within PINNs. While advanced models such as RNNs, LSTMs, and TCNs capture temporal dependencies more effectively, their substantial computational cost, combined with the already intensive training of PINNs, renders them unsuitable for large-scale experimentation. Therefore, this study focuses on more basic sequential network structures to balance modeling capacity with computational feasibility.

**Figure 3:**
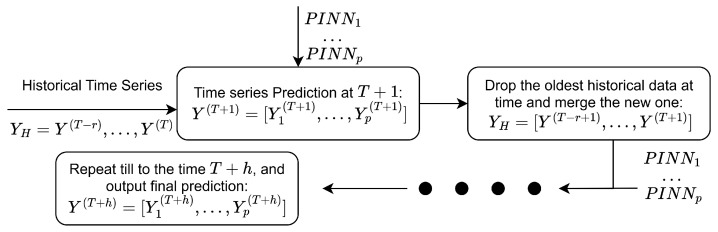
The prediction process for TV-VAR of PINN-based approach.

Finally, the prediction mechanism in our proposed framework for TV-VAR and TV-AR models fundamentally departs from that of conventional deep learning approaches. As mentioned earlier, instead of directly forecasting future time series values, the model is designed to estimate the time-varying parameters, the intercept terms $C^{\left(t\right)}$ and the coefficient matrices $B^{\left(t\right)}$, which govern the underlying dynamics. To predict the value at time T+h in the TV-VAR or TV-AR model with *r* lags, the process involves three steps: (1) construct the lagged input vector using observed data from time T−r to *T*; (2) use this vector to estimate the time-varying parameters $B^{\left(t\right)}$ and $C^{\left(t\right)}$, and compute the time series value at time *T*; and (3) iteratively repeat steps (1) and (2) until reaching time T+h. In the multivariate setting, the TV-VAR model requires training *p* separate networks, one for each dimension of the time series, to fully recover the system’s evolution at each step. The overall prediction architecture is depicted in Figure 3.

## 4. Simulation

In this section, we present a series of simulation studies using synthetic time series data to assess the performance of the proposed methods in practical scenarios. Specifically, the data generation process for the first-order time-varying (vector) autoregressive model is formally defined as follows:

$$\begin{array}{cc} \; & TV-AR:\;y^{\left(t\right)}=c^{\left(t\right)}+f\left(t\right)y^{\left(t-i\right)}+W_{\varepsilon }\varepsilon^{\left(t\right)},\\ \; & TV-VAR:\;Y^{\left(t\right)}=C^{\left(t\right)}+F\left(t\right)Y^{\left(t-1\right)}+W_{\varepsilon }E^{\left(t\right)}, \end{array}$$

where in TV-AR, $c^{\left(t\right)}\in R$ denotes the time-varying intercept term, while f(t)∈R represents the time-varying autoregressive coefficient for the first lag at time *t*. The noise term ε∼N(0,σ) is assumed to follow a Gaussian distribution with zero mean and variance σ∈R. Similarly, in the TV-VAR model, $C^{\left(t\right)}\in R^{\left(p\times 1\right)}$ is the time-varying intercept vector, and $F\left(t\right)\in R^{p\times p}$ denotes the time-varying coefficient matrix for the first lag at time *t*. The multivariate noise term E∼N(0,Σ) follows a Gaussian distribution with zero mean and covariance matrix $\Sigma \in R^{p\times p}$. Subsequently, to evaluate the forecasting accuracy of the proposed PINN-based methods, the mean absolute error (MAE), as defined in Equation (10), is computed over a forecast horizon of length H=2.

(10) $$MAE\left(\hat{y},y\right)=\frac{1}{n}\sum_{i=1}^{n}\left|{\hat{y}}_{i}-y_{i}\right|.$$

Typically, in the GAM-based approach, time-varying coefficients were represented as linear combinations of predefined basis functions, which were estimated from historical data and then used to generate forecasts over the next *H* time steps; prediction accuracy was then evaluated by computing the corresponding forecast error. In contrast, the PINN-based method utilized past observations to train a neural network that directly maps temporal inputs to time-varying coefficients, which were subsequently employed for multi-step forecasting over the same horizon. Lastly, the OKS method assumed a time-invariant coefficient matrix over the forecast window, with predictions generated under this fixed-coefficient assumption and the associated errors evaluated accordingly. All algorithms, simulations, and empirical analyses in this study were implemented in Python 3.9.19, with the GAM- and OKS-based implementations adapted from the foundational work of [51], and the PINN-based method’s source code and datasets detailed in the Data Availability Statement.

### 4.1. Simulation Preparation

In the case of a smoothly evolving transition matrix or value, we distinguish between two settings, TV-AR and TV-VAR, each characterized by the following specific scenarios:

- Scenario 1 (TV-AR): Consider a univariate time series with a lag order of r=1, and an initial value $y_{0}\in \left[-10,10\right]$ sampled uniformly at random. The additive noise is modeled as Gaussian with zero mean and variance σ=1, scaled by a noise weight factor $W_{\varepsilon }=1/10$. The time-varying autoregressive coefficient is initialized with a random scalar $a_{0}\in \left[0,1\right]$, and evolves over time according to the update rule:
  $$f\left(t\right)\leftarrow a_{0}-g\left(t\right),$$
  where the time-varying term g(t) is defined as $g\left(t\right)=t/\left(1+e^{-10(t-0.5)}\right)$, which induces a smooth, sigmoid-like transition in the coefficient matrix entries over time. Additionally, the intercept vector is defined as $c^{\left(t\right)}\leftarrow t-0.5$, ensuring that the system’s dynamics evolve continuously over a short time window, with gradual shifts between high and low coefficient values.
- Scenario 2 (TV-AR): The setting of this scenario is similar to TV-VAR Scenario 1, by introducing quadratic changes in the time-varying coefficients instead of sigmoid-like transitions. Specifically, the temporal perturbation term is defined as $g\left(t\right)={\left(t-0.5\right)}^{2}$, enabling evaluation of the performance of the model under non-stationary quadratic regimes.
- Scenario 3 (TV-VAR): Consider a multivariate time series with dimensionality p=2, lag order r=1, and an initial value $y_{0}\in \left[-10,10\right]$ sampled uniformly at random. The additive noise is modeled as Gaussian with zero mean and covariance matrix $\Sigma =diag\left(\vec{1}\right)\in R^{p\times p}$, and scaled by the noise weight $W_{\varepsilon }=1/10$. The time-varying coefficient matrix is initialized as a random matrix $A_{0}$, normalized row-wise to form a row-stochastic matrix, and updated using the following functions:
  $$F\left(t\right)\leftarrow \left[\begin{array}{cc} a_{0,0}^{\left(t-1\right)}+g\left(t\right), & 1-a_{0,0}^{\left(t\right)}\\ a_{1,0}^{\left(t-1\right)}-g\left(t\right), & 1-a_{1,0}^{\left(t\right)} \end{array}\right]$$
  where the time-varying perturbation term g(t) is defined as $g\left(t\right)=1/\left(1+e^{-10(t-0.5)}\right)$, mirroring the structure used in Scenario 1 but with a smaller intercept vector specified as $C^{\left(t\right)}\leftarrow \left(t-0.5\right)/10$, enabling gradual regime shifts over time.
- Scenario 4 (TV-VAR): The setting of this scenario is similar to TV-VAR Scenario 3, but replaces the sigmoid-like time-varying component g(t) with a quadratic structure, similarly to the one in Scenario 2.

Next, the L-BFGS-B algorithm from the SciPy optimization suite, a limited-memory quasi-Newton method known for its efficiency and fast convergence, was employed in the PINN-based approach, while the GMA- and OKS-based methods utilized the ‘trust-constr’ algorithm, which is also from SciPy and built on a constrained optimization framework using Lagrangian gradient descent. Both algorithms are well suited for efficiently handling large-scale optimization problems, particularly those arising in high-dimensional PINNs training. Finally, to rigorously evaluate the performance of the PINN-based model, we applied it across Scenarios 1 through 4 for full-horizon reconstruction with lag r=1, using the GAM-based approach as a baseline. Both models were trained on 95% of each synthetic time series comprising 200 data points, where the first 190 samples were used for model fitting and the remaining 10 were reserved for out-of-sample validation. After training, recursive multi-step prediction was performed: the model uses one initial lagged input to iteratively forecast the next point, updating the input window with each predicted value. This autoregressive roll-forward strategy continued until all 199 steps were reconstructed, enabling pointwise comparison against ground truth across the full temporal span. In contrast, the OKS-based and stationary VAR approaches were omitted from this analysis due to their inherent reliance on (local) stationarity assumptions, which constrain their capacity to capture the time-varying dynamics exhibited in these scenarios. All experiments were conducted on a workstation powered by an NVIDIA RTX 3050 Ti GPU and an AMD Ryzen 7 5800H CPU, utilizing GPU acceleration for batched matrix computations and neural network inference to significantly enhance training efficiency.

### 4.2. Simulation Results

In this research, to maximize flexibility in the GAM- and OKS-based methods, a diverse set of basis functions was selected. Specifically, these include linear and quadratic terms to capture basic polynomial trends; hyperbolic tangent (tanh) and exponential functions to model saturation effects and exponential growth; Gaussian functions for localized, smooth approximations; and cosine functions to represent periodic or oscillatory patterns. This rich combination of basis functions enhances the expressive capacity of the models, enabling them to adapt to a wide range of underlying functional forms in the estimation of time-varying coefficients. Next, for the PINN-based approach, a relatively simple network architecture was employed in this study. In particular, the model consists of three hidden layers with 10 neurons each, using the hyperbolic tangent (tanh) activation function, and it is trained for a maximum of 800 iterations.

**Figure 4:**
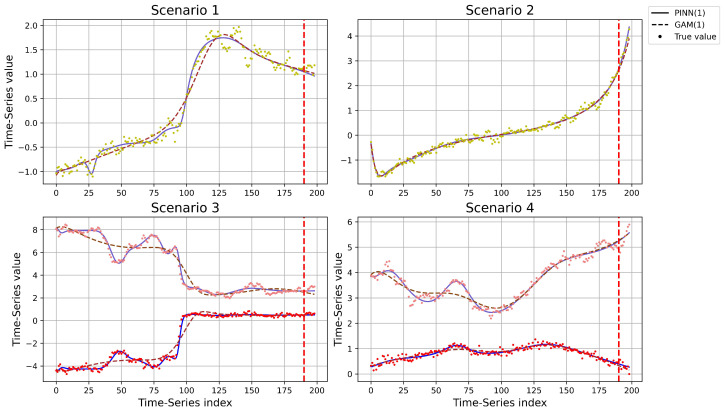
The reconstruction results of the GAM- and PINN-based methods were obtained using the first 190 time points for training and the remaining 10 for prediction, considering both one-dimensional and two-dimensional time series.

Finally, for each scenario, the predictions were made two steps ahead using 50 randomly generated time series samples, each of length 200, with time *t* normalized from 0 to 1, and the performance was evaluated using MAE across these steps. In this study, all models were estimated using lag orders r=1,2,3. The results for the two TV-AR scenarios are presented in Table 1 and Table 2, while the outcomes for the TV-VAR scenarios are shown in Table 3 and Table 4. Each table reports the mean absolute error (MAE) and the root sample variance (RSV) of absolute error values for each scenario. Finally, the reconstructed results of the GAM- and PINN-based method are shown in Figure 4.

### 4.3. Discussion

The simulation results compare the predictive performance of the VAR, GAM-, OKS-, and PINN-based methods in modeling TV-AR and TV-VAR processes across four scenarios. From the results, for H=1, the PINN-based method achieves the lowest MAE in Scenarios 1 and 4, while the OKS-based method performs best in Scenarios 2 and 3; for H=2, the PINN-based method again performs best in Scenarios 1, 2, and 4, with the GAM-based method achieving the lowest MAE in Scenario 3. Interestingly, the traditional VAR model does not consistently yield the worst results, especially in higher-dimensional settings, despite the time-varying and non-stationary nature of the processes. This is because the fixed coefficients in stationary models effectively approximate the average behavior of the time-varying coefficients over the entire time span, and when the variation is relatively small, the advantages of more complex non-stationary models, like GAM, OKS, or PINNs, become less significant. Also, as expected, in most scenarios, MAE generally increases as the forecast horizon *H* extends from 1 to 2, reflecting the accumulating uncertainty over time.

Additionally, we find that the performance of all non-stationary methods, including GAM-, OKS-, and PINN-based approaches, is generally insensitive to the length of the time series. In our experiments, series lengths of 200, 300, and 400 were tested, and the differences in MAE across these lengths were not significant. This insensitivity is likely due to the short forecasting horizon, a fixed two-step-ahead prediction, which limits temporal dependency and thereby reduces the influence of the overall input length on predictive accuracy. However, when the time series length decreases below 50, the PINN-based method occasionally exhibits underfitting, likely due to insufficient training data relative to its complex network architecture, characterized by multiple hidden layers and nonlinear activation functions, which demands a substantial sample size to properly optimize the high-dimensional parameter space and avoid poor local minima. Similar data limitations affect the GAM- and OKS-based methods, particularly in high-dimensional settings, where accurately estimating time-varying coefficients requires larger datasets to ensure reliable smoothing, prevent overfitting, and maintain stable convergence of the estimation algorithms. Therefore, as long as the time series length is sufficiently large, performance remains stable across different lengths.

In terms of computational and time complexity, the PINN-based approach exhibits significantly higher demands than the GAM- and OKS-based methods due to the intricate architecture of neural networks. Specifically, the relative complexity of the four frameworks can be summarized as follows: PINNs > OKS > GAM > VAR. The OKS method incurs greater computational cost than GAM primarily because of the cross-validation required for bandwidth selection, which effectively increases the number of evaluations rather than the number of model parameters. To assess the computational efficiency of the PINN-, GAM-, and OKS-based methods across varying time series dimensions, each method was executed 50 times per dimension under Scenario 1 and 3. The average processing times are reported in Table 5. For illustration, to illustrate this with an example, consider a TV-AR model with lag r=1, using five basis functions in both the GAM- and OKS-based approaches, and a neural network architecture with two hidden layers, each containing five neurons. In the GAM-based method, there are 10 parameters to estimate: 5 for the time-varying coefficients and 5 for the intercept. In the OKS-based method, only six parameters are estimated: five for the time-varying coefficients and one for the constant intercept. In contrast, the PINN framework, where each layer output is computed as y=f(Wx+b), results in a total of 52 trainable variables: 10 from the input to the first hidden layer (5 weights and 5 biases), 30 from the first to second hidden layer (25 weights and 5 biases), and 12 from the second hidden layer to the output (10 weights and 2 biases). Altogether, the network requires learning 52 parameters, highlighting the substantially greater parameter space and complexity of the PINN-based method.

Next, the PINN-based approach does not consistently deliver superior performance, primarily due to inherent challenges in training deep neural networks for time-varying systems, such as vanishing gradients, local minima, and sensitivity to initialization, which often result in overfitting or underfitting when the network architecture or hyperparameter configuration is suboptimal. In our experiments, we observed that underperforming cases could be significantly improved through careful architectural and training refinements, underscoring the model’s sensitivity to design choices. Critical hyperparameters influencing performance include the number of neurons per hidden layer, network depth, type and smoothness of activation functions, learning rate, and the choice of optimization algorithms. These considerations are particularly important in the context of modeling complex, time-varying dynamics, where the expressive capacity of the network must be carefully balanced against the risk of overparameterization and training instability.

Finally, when reconstructing the full trajectory of time-varying processes, neither VAR nor OKS prove to be adequate due to their foundational reliance on stationarity assumptions; VAR presumes global stationarity across the entire time horizon, while OKS is constrained by a local stationarity framework, typically relying on windowed estimation. As a result, these methods are inherently limited to pointwise prediction or short-horizon simulations and are incapable of accurately capturing the evolving temporal structure across the full process. In contrast, the PINN-based approach demonstrates a markedly superior reconstruction capability compared to the GAM-based method, particularly in scenarios characterized by nonlinear coefficient variation. These findings underscore the potential of PINN-based architectures for accurately reconstructing complex, non-stationary systems where conventional methods fail to capture global temporal dependencies.

## 5. Real-World Applications

In this real-world application, we investigate time series data within the context of health economics, specifically analyzing the dynamic relationship between unemployment rates and drug overdose deaths. Employment status is a well-established social determinant of health, which is closely linked to psychological distress, increased substance use, and higher mortality rates. Especially during periods of economic instability, individuals may adopt maladaptive coping mechanisms, such as drug misuse, which in turn can lead to elevated overdose deaths. Empirical studies support this connection: for example, research by the authors of [52] showed that a one-percentage-point rise in unemployment significantly increased opioid-related mortality at the county level. Similar patterns have been reported, especially during periods of economic downturn [53,54]. Despite substantial evidence, most existing research relies on annual or cross-sectional data and assumes a static, time-invariant relationship between unemployment and overdose deaths, even within panel data frameworks where the unemployment effect is typically modeled as constant over time [55,56]. While some studies account for nonlinearity or policy heterogeneity [57], very limited work has explored how this relationship evolves on a month-to-month basis. This highlights a notable gap in the literature, a lack of clear understanding of how short-term fluctuations in labor market conditions influence overdose mortality, particularly during disruptive events such as the COVID-19 pandemic. To address this gap, we apply a PINN-based time-varying autoregressive framework, using GAM-based, OKS-based, and traditional VAR models as benchmarks for comparison.

### 5.1. Data Preparation

This study utilizes a dataset spanning 60 months, from January 2020 to December 2024, across three regions: the District of Columbia, Maryland, and Virginia, resulting in a total of 180 observations (60 months × 3 regions). The dataset comprises two key variables, monthly unemployment rates from the U.S. Bureau of Labor Statistics and monthly drug overdose death counts from the CDC’s National Center for Health Statistics, capturing both urban and rural contexts as well as variations in public health and labor systems. By leveraging high-frequency data and flexible modeling techniques, we examine whether unemployment trends can serve as predictors of future overdose deaths. The findings aim to inform the development of early warning systems and support evidence-based policymaking at the local level.

### 5.2. Results

**Figure 5:**
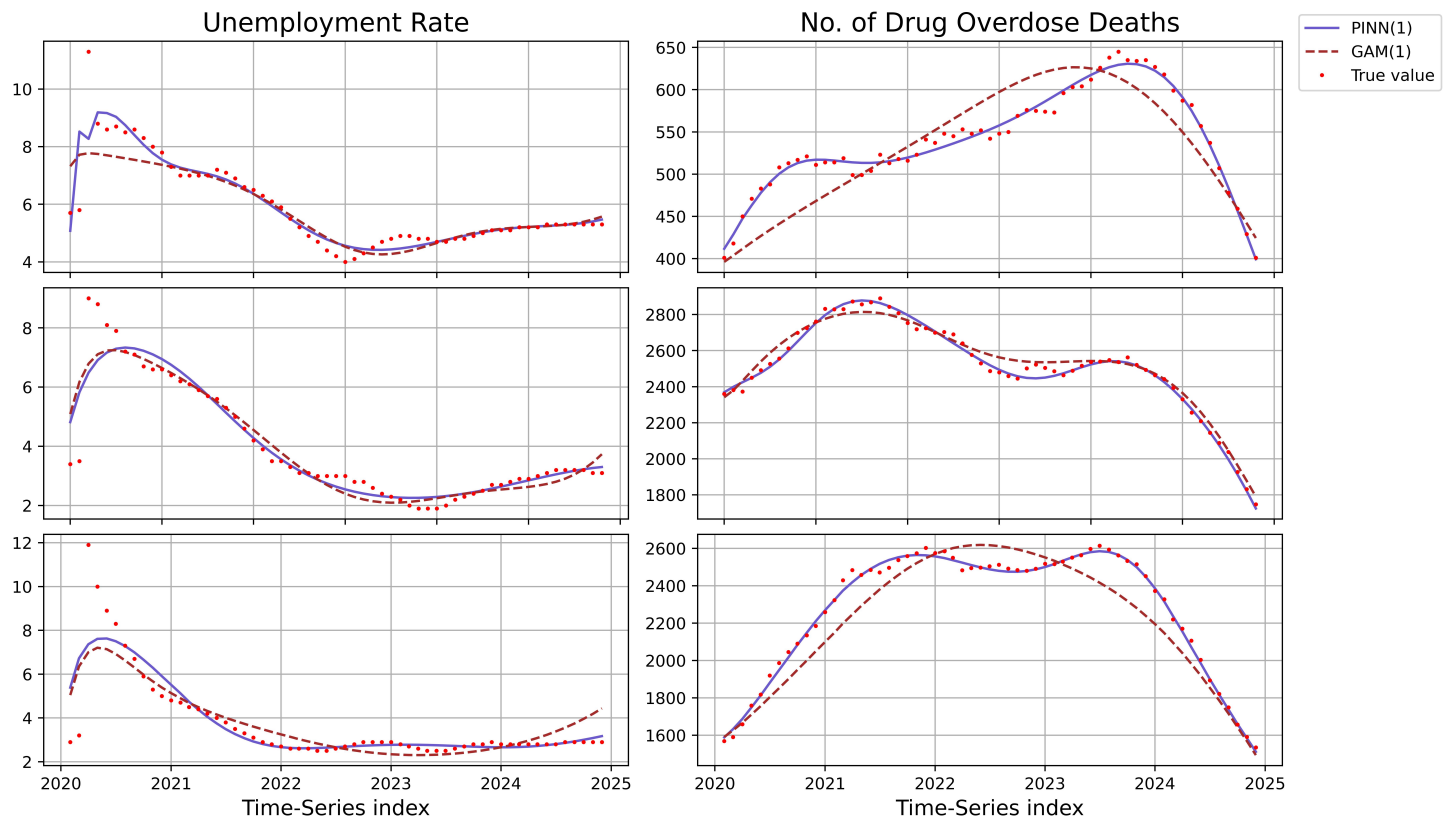
The reconstruction results of the GAM- and PINN-based methods in drug overdose data within three districts.

To assess the effectiveness of our proposed methods on real-world health data, we trained the TV-VAR model under all frameworks using data from 1 January 2020 to 1 November 2024, with lag orders r=1,2. The trained models were subsequently used to predict values for 1 December, using the PINN framework with a ‘tanh’ activation function, neural network architectures containing 5 and 10 neurons per layer, and a maximum number of iterations ranging from 800 to 1200. The predicted results were compared against the actual observations $y_{true}$, with absolute errors reported for the unemployment rate (RU) and the number of drug overdose deaths (DODs), as summarized in Table 6. Furthermore, leveraging the GAM- and PINN-based approaches with lag r=1, we reconstructed the entire time series and compared the resulting trajectories against the ground truth, as illustrated in Figure 5.

Overall, the PINN-based approach exhibits strong accuracy and stability across all regions. The results indicate that all non-stationary models outperform the traditional VAR model in most scenarios, with the most pronounced improvement observed in the District of Columbia, particularly at lag r=1. However, increasing the lag order *r* does not lead to a consistent improvement in performance, a common outcome in time series analysis, where longer lag structures can capture more temporal dependencies but also introduce greater model complexity and risk of overfitting. Furthermore, in reconstructing the full temporal trajectory, the PINN-based method more effectively captures the underlying dynamics than the GAM-based approach, especially in modeling the progression of drug overdose death counts.

Our results suggest that the unemployment rate is a useful predictor of near-term overdose mortality, although the strength and form of this relationship vary over time and between regions. The time-varying autoregressive framework allows us to capture short-term dynamics and shifts in the relationship between unemployment and overdose deaths. This adaptability is especially important during events such as COVID-19, when traditional models may not reflect the evolving economic and social pressures linked to overdose mortality. Although the model does not establish causality, it offers an adaptive tool for interpreting how labor market fluctuations may signal public health risks in real time.

This advantage likely stems from the limitations of the GAM framework, which heavily depends on predefined smooth basis functions. While GAMs are well suited for capturing gradual trends, their basis expansions can struggle with abrupt shifts or complex nonlinear patterns, leading to reduced fidelity in highly dynamic or structurally changing time series. In contrast, the PINN framework benefits from incorporating domain-specific constraints, enabling it to model complex system behaviors with greater precision and interpretability.

## 6. Conclusions

The Autoregressive (AR) model captures temporal dependencies through lagged linear terms but is limited by its assumption of stationarity, a constraint also present in its multivariate extension, the Vector Autoregressive (VAR) model, which assumes time-invariant parameters. Unfortunately, real-world applications in fields such as finance, climate science, healthcare, and signal processing often involve non-stationarity, and neglecting this evolving behavior can significantly degrade forecasting accuracy, underscoring the importance of Time-Varying (vector) Autoregressive (TV-AR/TV-VAR) models. Recent research shows that while Generalized Additive Models (GAMs) and Kernel Smoothing (KS) effectively capture dynamic relationships, their dependence on fixed basis functions or bandwidths limits adaptability and increases sensitivity to model selection, particularly amid nonlinearities, structural breaks, or hidden confounders. From an interpretability perspective, GAM- and KS-based models offer transparent decompositions of dynamic effects, yet their limited function spaces often fail to capture sharp transitions or high-frequency components, leading to underfitting, bias in derivative estimation, and suboptimal out-of-sample generalization. Furthermore, manually specifying basis functions introduces risks of model misspecification, particularly in high-dimensional settings involving complex lagged interactions, where inappropriate choices can compromise stability, increase estimation variance, and reduce robustness to noise or regime shifts. To address these limitations, we propose a physics-informed neural network (PINN)-based framework that replaces explicit basis design with implicit function approximation via deep neural architectures, guided by time-varying autoregressive structures. This hybrid design allows for non-parametric modeling of time-varying coefficients while ensuring adherence to structural interpretability, enabling the model to capture nonlinear, multiscale, and latent dynamics without the need for manual basis specification.

In contrast to GAM- and OKS-based methods, the PINN-based framework offers significantly greater modeling flexibility and robustness across a wide range of hyperparameter and architecture configurations, making it well suited for complex, real-world time series applications. By leveraging the universal function approximation capabilities of neural networks, PINNs can capture intricate temporal patterns without requiring manually specified basis functions and often achieve high accuracy even with common activation functions (e.g., ReLU, tanh) and shallow architectures. Additionally, while both GAM- and PINN-based models can reconstruct full temporal trajectories, PINNs exhibit significantly superior performance in highly non-stationary or high-frequency regimes, where traditional smoothing techniques often underfit and lose temporal resolution, thereby enhancing convergence and adaptability in data-rich, dynamically evolving systems. Unfortunately, despite their strengths, PINNs inherit several challenges common to deep learning frameworks, particularly in time and memory consumption. Moreover, ensuring stable and accurate performance often necessitates moderate to large volumes of training data, since data scarcity in such cases can lead to underfitting or convergence instability. This challenge is further exacerbated in high-dimensional time series settings, where the proliferation of variables and lagged dependencies significantly expands the parameter space, thereby increasing the computational burden and demanding substantially more training time and data. Collectively, these factors constrain the scalability and efficiency of PINNs in real-time or resource-limited applications.

Finally, future research in time-varying autoregressive modelling could explore two promising directions. First, enhancing the neural network architecture: while this study employed a basic sequential network structure, integrating more sophisticated architectures, such as convolutional neural networks, LSTM, and recurrent neural networks, into the PINN framework could improve the model’s capacity to capture complex temporal dependencies inherent in time-varying autoregressive systems. Second, refining the design of the time-varying loss function: the current study used an unconstrained formulation for parameter estimation, but future work could incorporate structural constraints or regularization techniques to enforce desirable properties (e.g., smoothness or sparsity) in the time-varying coefficients, potentially improving both interpretability and robustness. Additionally, a key advantage of PINNs over conventional deep learning architectures lies in their integration of physical priors, which enhances interpretability and improves generalization in physics-constrained systems. However, classical PINNs are fundamentally designed for systems governed by PDEs or ODEs, and their reliance on well-defined governing equations and boundary conditions limits their applicability in scenarios where such physical priors are incomplete, uncertain, or unavailable. Therefore, another promising direction for future research is to broaden the applicability of PINNs by relaxing their strict reliance on explicit PDE/ODE formulations, besides the Time-Varying Autoregressive model, with the goal of preserving interpretability while incurring only minimal loss in predictive accuracy.

## Figures and Tables

**Table 1:** The summary statistics of absolute errors across 50 random samples for Scenario 1 (TV-AR).

Method	H	MAE	RSV	Method	H	MAE	RSV
PINN (1)	1	$1.132\times 10^{-1}$	$6.853\times 10^{-2}$	PINN (1)	2	$1.012\times 10^{-1}$	$6.467\times 10^{-2}$
PINN (2)	1	$7.896\times 10^{-2}$	$4.737\times 10^{-2}$	PINN (2)	2	$8.114\times 10^{-2}$	$7.593\times 10^{-2}$
PINN (3)	1	$5.932\times 10^{-2}$	$3.835\times 10^{-2}$	PINN (3)	2	$4.222\times 10^{-2}$	$3.216\times 10^{-2}$
GAM (1)	1	$1.780\times 10^{-1}$	$8.089\times 10^{-2}$	GAM (1)	2	$2.740\times 10^{-1}$	$3.699\times 10^{-1}$
GAM (2)	1	$1.824\times 10^{-1}$	$8.824\times 10^{-2}$	GAM (2)	2	$2.748\times 10^{-1}$	$3.713\times 10^{-1}$
GAM (3)	1	$1.460\times 10^{-1}$	$8.507\times 10^{-2}$	GAM (3)	2	$2.623\times 10^{-1}$	$3.792\times 10^{-1}$
OKS (1)	1	$9.790\times 10^{-2}$	$4.411\times 10^{-2}$	OKS (1)	2	$1.071\times 10^{-1}$	$7.744\times 10^{-2}$
OKS (2)	1	$1.365\times 10^{-1}$	$9.445\times 10^{-2}$	OKS (2)	2	$8.529\times 10^{-2}$	$7.856\times 10^{-2}$
OKS (3)	1	$1.753\times 10^{-1}$	$1.325\times 10^{-1}$	OKS (3)	2	$4.579\times 10^{-2}$	$3.723\times 10^{-2}$
AR (1)	1	$1.523\times 10^{-1}$	$1.158\times 10^{-1}$	AR (1)	2	$1.677\times 10^{-1}$	$4.959\times 10^{-2}$
AR (2)	1	$1.751\times 10^{-1}$	$1.215\times 10^{-1}$	AR (2)	2	$1.640\times 10^{-1}$	$5.982\times 10^{-2}$
AR (3)	1	$1.435\times 10^{-1}$	$1.224\times 10^{-1}$	AR (3)	2	$1.647\times 10^{-1}$	$9.846\times 10^{-2}$

**Table 2:** The summary statistics of absolute errors across 50 random samples for Scenario 2 (TV-AR).

Method	H	MAE	RSV	Method	H	MAE	RSV
PINN (1)	1	$1.222\times 10^{-1}$	$5.249\times 10^{-2}$	PINN (1)	2	$1.748\times 10^{-1}$	$1.137\times 10^{-1}$
PINN (2)	1	$1.081\times 10^{-1}$	$6.006\times 10^{-2}$	PINN (2)	2	$1.239\times 10^{-1}$	$6.987\times 10^{-2}$
PINN (3)	1	$8.020\times 10^{-2}$	$6.898\times 10^{-2}$	PINN (3)	2	$8.873\times 10^{-2}$	$6.453\times 10^{-2}$
GAM (1)	1	$1.803\times 10^{-1}$	$9.305\times 10^{-2}$	GAM (1)	2	$2.741\times 10^{-1}$	$1.734\times 10^{-1}$
GAM (2)	1	$1.375\times 10^{-1}$	$1.013\times 10^{-1}$	GAM (2)	2	$2.588\times 10^{-1}$	$1.653\times 10^{-1}$
GAM (3)	1	$8.803\times 10^{-2}$	$5.455\times 10^{-2}$	GAM (3)	2	$1.718\times 10^{-1}$	$4.325\times 10^{-2}$
OKS (1)	1	$2.023\times 10^{-1}$	$1.743\times 10^{-1}$	OKS (1)	2	$4.185\times 10^{-1}$	$3.031\times 10^{-1}$
OKS (2)	1	$8.417\times 10^{-2}$	$4.625\times 10^{-2}$	OKS (2)	2	$2.242\times 10^{-1}$	$1.004\times 10^{-1}$
OKS (3)	1	$6.424\times 10^{-2}$	$3.438\times 10^{-2}$	OKS (3)	2	$1.674\times 10^{-1}$	$7.436\times 10^{-2}$
AR (1)	1	$2.726\times 10^{-1}$	$2.105\times 10^{-1}$	AR (1)	2	$3.122\times 10^{-1}$	$1.615\times 10^{-1}$
AR (2)	1	$2.162\times 10^{-1}$	$1.544\times 10^{-1}$	AR (2)	2	$2.223\times 10^{-1}$	$1.444\times 10^{-1}$
AR (3)	1	$1.235\times 10^{-1}$	$1.003\times 10^{-1}$	AR (3)	2	$1.157\times 10^{-1}$	$7.006\times 10^{-2}$

**Table 3:** The summary statistics of absolute errors across 50 random samples for Scenario 3 (TV-VAR).

Method	H	MAE	RSV	Method	H	MAE	RSV
PINN (1)	1	$1.205\times 10^{-1}$	$8.832\times 10^{-2}$	PINN (1)	2	$1.287\times 10^{-1}$	$9.379\times 10^{-2}$
PINN (2)	1	$1.305\times 10^{-1}$	$9.135\times 10^{-2}$	PINN (2)	2	$1.618\times 10^{-1}$	$1.185\times 10^{-1}$
PINN (3)	1	$1.398\times 10^{-1}$	$8.337\times 10^{-2}$	PINN (3)	2	$1.813\times 10^{-1}$	$1.153\times 10^{-1}$
GAM (1)	1	$1.681\times 10^{-1}$	$6.693\times 10^{-2}$	GAM (1)	2	$1.132\times 10^{-1}$	$1.025\times 10^{-1}$
GAM (2)	1	$2.213\times 10^{-1}$	$2.097\times 10^{-1}$	GAM (2)	2	$2.565\times 10^{-1}$	$5.317\times 10^{-1}$
GAM (3)	1	$1.964\times 10^{-1}$	$2.186\times 10^{-1}$	GAM (3)	2	$2.556\times 10^{-1}$	$5.317\times 10^{-1}$
OKS (1)	1	$1.234\times 10^{-1}$	$9.455\times 10^{-2}$	OKS (1)	2	$1.445\times 10^{-1}$	$9.688\times 10^{-2}$
OKS (2)	1	$1.271\times 10^{-1}$	$8.326\times 10^{-2}$	OKS (2)	2	$1.634\times 10^{-1}$	$1.087\times 10^{-1}$
OKS (3)	1	$1.164\times 10^{-1}$	$8.356\times 10^{-2}$	OKS (3)	2	$1.562\times 10^{-1}$	$1.057\times 10^{-1}$
VAR (1)	1	$1.222\times 10^{-1}$	$6.091\times 10^{-2}$	VAR (1)	2	$1.213\times 10^{-1}$	$1.064\times 10^{-1}$
VAR (2)	1	$1.291\times 10^{-1}$	$6.262\times 10^{-2}$	VAR (2)	2	$1.325\times 10^{-1}$	$1.122\times 10^{-1}$
VAR (3)	1	$1.450\times 10^{-1}$	$5.945\times 10^{-2}$	VAR (3)	2	$1.274\times 10^{-1}$	$1.060\times 10^{-1}$

**Table 4:** The summary statistics of absolute errors across 50 random samples for Scenario 4 (TV-VAR).

Method	H	MAE	RSV	Method	H	MAE	RSV
PINN (1)	1	$6.771\times 10^{-2}$	$5.234\times 10^{-2}$	PINN (1)	2	$1.029\times 10^{-1}$	$7.238\times 10^{-2}$
PINN (2)	1	$6.508\times 10^{-2}$	$5.427\times 10^{-2}$	PINN (2)	2	$1.178\times 10^{-1}$	$1.119\times 10^{-1}$
PINN (3)	1	$8.137\times 10^{-2}$	$6.326\times 10^{-2}$	PINN (3)	2	$1.128\times 10^{-1}$	$1.081\times 10^{-1}$
GAM (1)	1	$1.025\times 10^{-1}$	$9.080\times 10^{-2}$	GAM (1)	2	$1.219\times 10^{-1}$	$1.021\times 10^{-1}$
GAM (2)	1	$9.031\times 10^{-1}$	$9.295\times 10^{-2}$	GAM (2)	2	$1.275\times 10^{-1}$	$1.215\times 10^{-1}$
GAM (3)	1	$8.871\times 10^{-1}$	$9.284\times 10^{-2}$	GAM (3)	2	$1.358\times 10^{-1}$	$1.277\times 10^{-1}$
OKS (1)	1	$8.524\times 10^{-2}$	$6.761\times 10^{-2}$	OKS (1)	2	$1.866\times 10^{-1}$	$1.385\times 10^{-1}$
OKS (2)	1	$8.986\times 10^{-2}$	$6.021\times 10^{-2}$	OKS (2)	2	$1.624\times 10^{-1}$	$1.283\times 10^{-1}$
OKS (3)	1	$9.907\times 10^{-2}$	$5.713\times 10^{-2}$	OKS (3)	2	$1.480\times 10^{-1}$	$1.264\times 10^{-1}$
VAR (1)	1	$7.043\times 10^{-2}$	$4.183\times 10^{-2}$	VAR (1)	2	$1.315\times 10^{-1}$	$6.209\times 10^{-2}$
VAR (2)	1	$7.199\times 10^{-2}$	$4.528\times 10^{-2}$	VAR (2)	2	$1.422\times 10^{-1}$	$8.195\times 10^{-2}$
VAR (3)	1	$6.960\times 10^{-2}$	$4.467\times 10^{-2}$	VAR (3)	2	$1.224\times 10^{-1}$	$8.435\times 10^{-2}$

**Table 5:** Computational time for the PINN-, GAM-, and OKS-based methods across Scenarios 1 and 3.

Methods	Lags	Second	Lags	Second	Lags	Second
AR	1	0.35	2	0.48	3	0.66
GAM	1	3.51	2	4.56	3	8.78
OKS	1	10.10	2	17.06	3	22.76
PINN	1	68.11	2	68.04	3	68.63
VAR	1	0.77	2	0.33	3	0.21
GAM	1	7.88	2	10.68	3	12.83
OKS	1	56.66	2	48.66	3	37.10
PINN	1	136.92	2	130.05	3	130.86

**Table 6:** The summary statistics of real-world application.

Method	Location	AE (UR, r = 1)	AE (DOD, r = 1)	AE (UR, r = 2)	AE (DOD, r = 2)
PINN	DC	$9.817\times 10^{-2}$	1.679	$5.626\times 10^{-2}$	1.389
GAM	DC	$2.251\times 10^{-1}$	11.186	$4.538\times 10^{-2}$	6.338
OKS	DC	$3.919\times 10^{-2}$	3.682	$4.076\times 10^{-2}$	3.808
VAR	DC	$7.945\times 10^{-1}$	31.684	$5.867\times 10^{-1}$	16.194
PINN	MD	$3.565\times 10^{-2}$	10.032	$1.326\times 10^{-1}$	2.379
GAM	MD	$4.641\times 10^{-1}$	0.854	$5.364\times 10^{-1}$	6.571
OKS	MD	$7.932\times 10^{-2}$	22.108	$1.921\times 10^{-2}$	22.211
VAR	MD	$3.945\times 10^{-1}$	44.878	$2.001\times 10^{-1}$	4.316
PINN	VA	$2.712\times 10^{-1}$	4.723	$1.874\times 10^{-1}$	10.900
GAM	VA	$7.865\times 10^{-1}$	20.018	$8.157\times 10^{-1}$	33.030
OKS	VA	$4.632\times 10^{-2}$	11.285	$3.600\times 10^{-2}$	12.376
VAR	VA	1.364	10.886	1.543	8.307

## Data Availability

The data and code used in this study are available on GitHub at https://github.com/zhixuan1994/Time-varying-Vector-Autoregressive-Model-PINNs-based-approach-.git (accessed on 30 August 2025) for purposes of replication and further research. Additionally, they will be deposited in a publicly accessible repository upon publication to ensure transparency and facilitate broader access to the research community.

## References

[1] Scholten S., Rubel J.A., Glombiewski J.A., Milde C. (2025). What time-varying network models based on functional analysis tell us about the course of a patient’s problem. Psychother. Res..

[2] Neumann N.D., Yperen N.W.V., Arens C.R., Brauers J.J., Lemmink K.A.P.M., Emerencia A.C., Meerhoff L.A., Frencken W.G.P., Brink M.S., Hartigh R.J.R.D. (2025). How do psychological and physiological performance determinants interact within individual athletes? An analytical network approach. Int. J. Sport Exerc. Psychol..

[3] Hamilton J.D. (1994). Time Series Analysis.

[4] Jiang X.Q., Kitagawa G. (1993). A time varying coefficient vector AR modeling of nonstationary covariance time series. Signal Process..

[5] Cuomo S., Di Cola V.S., Giampaolo F., Rozza G., Raissi M., Piccialli F. (2022). Scientific Machine Learning Through Physics–Informed Neural Networks: Where we are and What’s Next. J. Sci. Comput..

[6] Haslbeck J.M.B., Waldorp L.J. (2020). mgm: Estimating Time-Varying Mixed Graphical Models in High-Dimensional Data. J. Stat. Softw..

[7] Raissi M., Perdikaris P., Karniadakis G. (2019). Physics-informed neural networks: A deep learning framework for solving forward and inverse problems involving nonlinear partial differential equations. J. Comput. Phys..

[8] Guo Y., Cao X., Liu B., Gao M. (2020). Solving Partial Differential Equations Using Deep Learning and Physical Constraints. Appl. Sci..

[9] Fazal F.U., Sulaiman M., Bassir D., Alshammari F.S., Laouini G. (2024). Quantitative Analysis of the Fractional Fokker–Planck–Levy Equation via a Modified Physics-Informed Neural Network Architecture. Fractal Fract..

[10] Cai S., Wang Z., Wang S., Perdikaris P., Karniadakis G.E. (2021). Physics-Informed Neural Networks for Heat Transfer Problems. J. Heat Transf..

[11] Tartakovsky A., Marrero C., Perdikaris P., Tartakovsky G., Barajas-Solano D. (2020). Physics-Informed Deep Neural Networks for Learning Parameters and Constitutive Relationships in Subsurface Flow Problems. Water Resour. Res..

[12] Ramos D.J., Cunha B.Z., Daniel G.B. (2023). Evaluation of physics-informed neural networks (PINN) in the solution of the Reynolds equation. J. Braz. Soc. Mech. Sci. Eng..

[13] Hou Q., Li Y., Singh V.P., Sun Z. (2024). Physics-informed neural network for diffusive wave model. J. Hydrol..

[14] Zhang D., Guo L., Karniadakis G.E. (2020). Learning in Modal Space: Solving Time-Dependent Stochastic PDEs Using Physics-Informed Neural Networks. SIAM J. Sci. Comput..

[15] CHEN X., DUAN J., KARNIADAKIS G.E. (2021). Learning and meta-learning of stochastic advection–diffusion–reaction systems from sparse measurements. Eur. J. Appl. Math..

[16] Yang L., Meng X., Karniadakis G.E. (2021). B-PINNs: Bayesian physics-informed neural networks for forward and inverse PDE problems with noisy data. J. Comput. Phys..

[17] Elhareef M.H., Wu Z. (2023). Physics-informed neural network method and application to nuclear reactor calculations: A pilot study. Nucl. Sci. Eng..

[18] Lee S., Popovics J. (2022). Applications of physics-informed neural networks for property characterization of complex materials. RILEM Tech. Lett..

[19] Wang D., Jiang X., Song Y., Fu M., Zhang Z., Chen X., Zhang M. (2022). Applications of Physics-Informed Neural Network for Optical Fiber Communications. IEEE Commun. Mag..

[20] Wang Y., Wei M., Dai F., Zou D., Lu C., Han X., Chen Y., Ji C. (2025). Physics-Informed Fractional-Order Recurrent Neural Network for Fast Battery Degradation with Vehicle Charging Snippets. Fractal Fract..

[21] Yang Y., Li H. (2025). Neural Ordinary Differential Equations for robust parameter estimation in dynamic systems with physical priors. Appl. Soft Comput..

[22] Nie F., Fang H., Wang J., Zhao L., Jia C., Ma S., Wu F., Zhao W., Yang S., Wei S. (2025). An Adaptive Solid-State Synapse with Bi-Directional Relaxation for Multimodal Recognition and Spatio-Temporal Learning. Adv. Mater..

[23] Ren D., Wang C., Wei X., Zhang Y., Han S., Xu W. (2025). Harmonizing physical and deep learning modeling: A computationally efficient and interpretable approach for property prediction. Scr. Mater..

[24] Yu J., Wang H., Chen M., Han X., Deng Q., Yang C., Zhu W., Ma Y., Yin F., Weng Y. (2024). A novel method to select time-varying multivariate time series models for the surveillance of infectious diseases. BMC Infect. Dis..

[25] Giudici P., Tarantino B., Roy A. (2023). Bayesian time-varying autoregressive models of COVID-19 epidemics. Biom. J..

[26] Maleki M., Bidram H., Wraith D. (2023). Robust clustering of COVID-19 cases across U.S. counties using mixtures of asymmetric time series models with time varying and freely indexed covariates. J. Appl. Stat..

[27] Azhar M.A.R., Adi Nugroho H., Wibirama S. The Study of Multivariable Autoregression Methods to Forecast Infectious Diseases. Proceedings of the 2021 IEEE 5th International Conference on Information Technology, Information Systems and Electrical Engineering (ICITISEE).

[28] Ding F., Xu L., Liu P., Wang X. (2025). Two-stage parameter estimation methods for linear time-invariant continuous-time systems. Syst. Control Lett..

[29] Ji Y., Jiang A. (2023). Filtering-Based Accelerated Estimation Approach for Generalized Time-Varying Systems With Disturbances and Colored Noises. IEEE Trans. Circuits Syst. II Express Briefs.

[30] Ji Y., Liu J., Liu H. (2023). An identification algorithm of generalized time-varying systems based on the Taylor series expansion and applied to a pH process. J. Process Control.

[31] Zhao Y., Ji Y. (2025). Weighted multi-innovation parameter estimation for a time-varying Volterra–Hammerstein system with colored noise. Optim. Control Appl. Methods.

[32] Sato J.R., Morettin P.A., Arantes P.R., Amaro E. (2007). Wavelet based time-varying vector autoregressive modelling. Comput. Stat. Data Anal..

[33] Liu Z., Wang L., Xu S., Lu K. (2023). A multiwavelet-based sparse time-varying autoregressive modeling for motor imagery EEG classification. Comput. Biol. Med..

[34] Bringmann L.F., Hamaker E.L., Vigo D.E., Aubert A., Borsboom D., Tuerlinckx F. (2017). Changing dynamics: Time-varying autoregressive models using generalized additive modeling. Psychol. Methods.

[35] Bringmann L.F., Ferrer E., Hamaker E.L., Borsboom D., Tuerlinckx F. (2018). Modeling Nonstationary Emotion Dynamics in Dyads using a Time-Varying Vector-Autoregressive Model. Multivar. Behav. Res..

[36] Ren B., Lucey B. (2023). Herding in the Chinese renewable energy market: Evidence from a bootstrapping time-varying coefficient autoregressive model. Energy Econ..

[37] Coronado S., Martinez J.N., Romero-Meza R. (2022). Time-varying multivariate causality among infectious disease pandemic and emerging financial markets: The case of the Latin American stock and exchange markets. Appl. Econ..

[38] Mohamad A., Inani S.K. (2023). Price discovery in bitcoin spot or futures during the Covid-19 pandemic? Evidence from the time-varying parameter vector autoregressive model with stochastic volatility. Appl. Econ. Lett..

[39] Zou Y., Chen Q., Han J., Xiao M. (2025). Measuring the Risk Spillover Effect of RCEP Stock Markets: Evidence from the TVP-VAR Model and Transfer Entropy. Entropy.

[40] Sheng D., Wang D., Zhang J., Wang X., Zhai Y. (2024). A Time-Varying Mixture Integer-Valued Threshold Autoregressive Process Driven by Explanatory Variables. Entropy.

[41] Zhang W., Lin Z., Liu X. (2022). Short-term offshore wind power forecasting—A hybrid model based on Discrete Wavelet Transform (DWT), Seasonal Autoregressive Integrated Moving Average (SARIMA), and deep-learning-based Long Short-Term Memory (LSTM). Renew. Energy.

[42] Wang H., Zhang Y., Liang J., Liu L. (2023). DAFA-BiLSTM: Deep Autoregression Feature Augmented Bidirectional LSTM network for time series prediction. Neural Netw..

[43] Laurenti L., Tinti E., Galasso F., Franco L., Marone C. (2022). Deep learning for laboratory earthquake prediction and autoregressive forecasting of fault zone stress. Earth Planet. Sci. Lett..

[44] Nasir J., Aamir M., Haq Z.U., Khan S., Amin M.Y., Naeem M. (2023). A New Approach for Forecasting Crude Oil Prices Based on Stochastic and Deterministic Influences of LMD Using ARIMA and LSTM Models. IEEE Access.

[45] Luo K., Zhao J., Wang Y., Li J., Wen J., Liang J., Soekmadji H., Liao S. (2025). Physics-informed neural networks for PDE problems: A comprehensive review. Artif. Intell. Rev..

[46] Rohrhofer F.M., Posch S., Gößnitzer C., Geiger B.C. (2023). Data vs. Physics: The Apparent Pareto Front of Physics-Informed Neural Networks. IEEE Access.

[47] Barimah A.K., Onu O.P., Niculita O., Cowell A., McGlinchey D. (2025). Scalable Data Transformation Models for Physics-Informed Neural Networks (PINNs) in Digital Twin-Enabled Prognostics and Health Management (PHM) Applications. Computers.

[48] Fernández de la Mata F., Gijón A., Molina-Solana M., Gómez-Romero J. (2023). Physics-informed neural networks for data-driven simulation: Advantages, limitations, and opportunities. Phys. A Stat. Mech. Its Appl..

[49] Chen C., Tang L., Lu Y., Wang Y., Liu Z., Liu Y., Zhou L., Jiang Z., Yang B. (2023). Reconstruction of long-term strain data for structural health monitoring with a hybrid deep-learning and autoregressive model considering thermal effects. Eng. Struct..

[50] Ghazi M.M., Sørensen L., Ourselin S., Nielsen M. (2024). CARRNN: A Continuous Autoregressive Recurrent Neural Network for Deep Representation Learning From Sporadic Temporal Data. IEEE Trans. Neural Netw. Learn. Syst..

[51] Jia Z., Li W., Jiang Y., Liu X. (2025). The Use of Minimization Solvers for Optimizing Time-Varying Autoregressive Models and Their Applications in Finance. Mathematics.

[52] Hollingsworth A., Ruhm C.J., Simon K. (2017). Macroeconomic conditions and opioid abuse. J. Health Econ..

[53] Ruhm C.J. (2000). Are recessions good for your health?. Q. J. Econ..

[54] Macmadu A., Batthala S., Gabel A.M.C., Rosenberg M., Ganguly R., Yedinak J.L., Hallowell B.D., Scagos R.P., Samuels E.A., Cerdá M. (2021). Comparison of characteristics of deaths from drug overdose before vs during the COVID-19 pandemic in Rhode Island. JAMA Netw. Open.

[55] Martins S.S., Segura L.E., Marziali M.E., Bruzelius E., Levy N.S., Gutkind S., Santarin K., Sacks K., Fox A. (2024). Higher unemployment benefits are associated with reduced drug overdose mortality in the United States before and during the COVID-19 pandemic. Int. J. Drug Policy.

[56] Wu P., Evangelist M. (2022). Unemployment insurance and opioid overdose mortality in the United States. Demography.

[57] Casal B., Iglesias E., Rivera B., Currais L., Storti C.C. (2023). Identifying the impact of the business cycle on drug-related harms in European countries. Int. J. Drug Policy.

